# Assessing the Roles of Molecular Markers of Antimalarial Drug Resistance and the Host Pharmacogenetics in Drug-Resistant Malaria

**DOI:** 10.1155/2022/3492696

**Published:** 2022-05-17

**Authors:** Peter Hodoameda, Nancy Odurowah Duah-Quashie, Neils Ben Quashie

**Affiliations:** ^1^West African Center for Cell Biology of Infectious Pathogens, University of Ghana, P. O. Box LG54, Legon, Ghana; ^2^Department of Epidemiology, Noguchi Memorial Institute for Medical Research, College of Health Sciences, University of Ghana, P. O. Box LG581, Legon, Ghana; ^3^Centre for Tropical Clinical Pharmacology and Therapeutics, University of Ghana Medical School, P. O. Box GP4236, Accra, Ghana

## Abstract

Malaria caused by the *Plasmodium* parasites is a major public health concern in malaria-endemic regions with *P. falciparum* causing the most severe form of the disease. The use of antimalarial drugs for the management of the disease proves to be one of the best methods to manage the disease. Unfortunately, *P. falciparum* has developed resistance to almost all the current in-use antimalarial drugs. Parasite development of resistance is primarily caused by both parasite and host genetic factors. The parasite genetic factors involve undergoing mutation in the drug target sites or increasing the drug target gene copy number to prevent the intended action of the antimalarial drugs. The host pharmacogenetic factors which determine how a particular antimalarial drug is metabolized could result in variations of drug plasma concentration and consequently contribute to variable treatment outcomes and the emergence or propagation of resistant parasites. Since both host and parasite genomes play a role in antimalarial drug action, a key question often asked is, “which of the two strongly drives or controls antimalarial drug resistance?” A major finding in our recent study published in the Malaria Journal indicates that the parasite's genetic factors rather than the host are likely to energize resistance to an antimalarial drug. However, others have reported contrary findings suggesting that the host genetic factors are the force behind resistance to antimalarial drugs. To bring clarity to these observations, there is the need for deciphering the major driving force behind antimalarial drug resistance through optimized strategies aimed at alleviating the phenomenon. In this direction, literature was systematically reviewed to establish the role and importance of each of the two factors aforementioned in the etiology of drug-resistant malaria. Using Internet search engines such as Pubmed and Google, we looked for terms likely to give the desired information which we herein present. We then went ahead to leverage the obtained information to discuss the globally avid aim of combating antimalarial drug resistance.

## 1. Introduction

Antimalaria drug resistance (ADR) continues to hinder global efforts to effectively manage and eradicate malaria disease [1, 2]. So far, of the *Plasmodium* species known to infect humans, *P. falciparum* has developed resistance to almost all antimalarials used for malaria treatment. ADR in the *P. falciparum* is known to emerge from low-transmission regions and spread to high-transmission areas [2, 3]. Parasite strains resistant to chloroquine (CQ) and sulfadoxine-pyrimethamine (SP) emerged from Southeast Asia (SEA) or South America before spreading to sub-Saharan Africa (sSA) [4, 5].

The high prevalence of CQ-resistant and SP-resistant parasites necessitated the introduction of artemisinin-based combination therapy (ACT) for the treatment of uncomplicated malaria in malaria-endemic regions. The highly efficacious ACT regimens were quickly adopted by most malaria-endemic countries as their first-line treatment option for uncomplicated malaria [6]. Unfortunately, partial resistance to the artemisinin (ART) component of the ACT, which is defined as “slower clearance of malaria parasitemia in the first 3 days of ART monotherapy or ACT treatment,” was reported in the western part of Cambodia in 2008 and 2009 [7, 8] and in the Greater Mekong Subregion [9–11]. This situation is a setback to the efficacy of the ACT regimens and consequential to the management of malaria. These concerns have subsequently been aggravated by the selection of parasites with partial resistance to the ART partner drug(s). Reports of treatment failures with dihydroartemisinin-piperaquine (DHAP) in Cambodia [12–14] and artesunate-mefloquine (ASMQ) on the Thai-Myanmar border [15] support this assertion.

The early detection of resistant parasite strains is crucial in the fight against malaria, as it will allow prompt identification and containment of these resistant strains. For the early detection of resistant parasite strains to a particular antimalarial drug, there is the need to understand the mechanisms at play in *Plasmodium* spp. antimalarial drug resistance development [16].

Certain mutation in the parasite genome confers resistance to certain antimalarial drugs. Malaria treatment failure is not only dependent on drug-resistant *P. falciparum* bearing these mutations but also on other factors such as incorrect use or suboptimum drug dosage, noncompliance to a drug regimen, use of counterfeit or fake drugs, drug-drug interactions [17], and poor drug metabolism [18]. Suboptimal drug concentration in blood contributes to poor malaria treatment outcomes leading to the emergence and/or spread of parasite-resistant strains [18]. On the other hand, a high drug concentration in blood is more likely to result in increased toxicity. The pharmacokinetic profile of a drug (absorption, distribution, metabolism, and excretion) can differ substantially among individuals with different cytochrome (CYP) genes. These make the drug metabolism enzymes (e.g., cytochrome P450 enzymes) and transport proteins (e.g., P-glycoproteins) very important in the breakdown, absorption, distribution, and excretion of antimalarial drugs [19].

The genetic variations in the genes encoding these enzymes in an individual may be responsible for differences in individual responses to antimalarial drugs. This suggests that it is important to consider the pharmacogenetics of individual patients before administering any particular antimalarial drug [18, 20].

This evidence shows that the most important factors that are principal in determining the efficacy of antimalarial drugs are the parasite genetic factors and pharmacogenetics [3, 18, 21]. Hence, this review aims to highlight the parasite genetic factors and host pharmacogenetic factors that could affect the efficacy of an antimalarial drug and attempts to leverage this towards the management of antimalarial drug resistance.

## 2. Malaria: A Brief Account of the Current Situation

The World Health Organization (WHO) reported 241 million cases of malaria worldwide in 2020 [22]. This indicates a decline in cases compared to the 251 million malaria cases reported in 2010 and an increase in cases compared to the 231 million cases reported in 2017. The WHO African Region recorded 228 million malaria cases out of the total 241 million malaria cases in 2020, representing 95% of the total malaria cases. This was followed by the WHO Southeast Asia Region, which recorded 3% of all malaria cases [22]. The WHO Eastern Mediterranean Region recorded 2% of the malaria cases recorded in 2020 [22].

## 3. Molecular Markers of Antimalarial Drug Resistance

The use of molecular markers of resistance to monitor the emergence and spread of parasites resistant to antimalarial drugs proves to be a very effective method in monitoring ADR [2]. The identification and validation of these molecular markers have boosted our confidence in using these tools to monitor ADR in malaria-endemic areas [2]. Markers such as mutations in the *P. falciparum* chloroquine resistance transporter gene (pfcrt) [23], *P. falciparum* multidrug resistance protein 1 gene (pfmdr1) [24], and *P. falciparum* kelch 13 gene (pfk13) [25] have been linked to resistance to CQ, lumefantrine (LMF), and ART, respectively. The underlining mechanisms of *Plasmodium* spp. resistance to these antimalarial drugs include undergoing mutations in the parasite genome resulting in changing the original transporter protein conformation which leads to expelling the drug from the digestive vacuole at a faster rate, loss of binding affinity between the drug and its target, or increased in gene copy number in the case of pfmdr1 [26–28].

## 4. Cross-Resistance of *P. falciparum* to Antimalarial Drugs

*P. falciparum* has developed cross-resistance to some antimalarial drugs that are in the same class, chemically related, and/or have a similar mechanism of action. The development of resistance to one antimalarial drug can set the right precedent for the development of resistance to other antimalarial drugs [29]. Cross-resistance has been reported for two 4-aminoquinolines drugs, that is, amodiaquine and chloroquine. Cross-resistance to amodiaquine and chloroquine has been reported in both clinical and laboratory isolates. For the quinoline drugs, cross-resistance has been reported between MQ, QN, and HLF. There are high cases of cross-resistance reported between HLF and MQ, especially in MQ-resistant clinical isolates [30]. Cross-resistance has also been recorded between LMF and MQ, which is caused by a mutation in pfmdr1 N86Y [31]. In a few cases, resistance to one drug confers increased susceptibility to other drugs. For example, pfmdr1 N86Y causes decreased susceptibility to CQ but increased susceptibility to MQ, while the increased pfmdr1 copy number is associated with increased CQ sensitivity and decreased MQ susceptibility [32]. In antifolates, cross-resistance has been observed between cycloguanil and pyrimethamine [33].

## 5. Controversies Surrounding the Use of Molecular Markers in the Surveillance of Resistant Parasite Strain

The use of molecular markers of resistance to monitor the emergence and spread of parasite strains resistant to antimalarial drugs has proven to be very effective. Nonetheless, this comes with its challenges, especially when there is a lack of universality in a particular molecular marker of resistance used for monitoring ADR. For example, the major mutations that have been reported as molecular markers of resistance to ART and its derivatives in SEA are pf k13 C580Y, R539T, and Y493H [34], but this is not the case in most African countries. This could probably be due to low levels of resistance to ART in most African countries. In cases with delayed ART treatment outcomes in most African countries, pfk13 C580Y, R539T, and Y493H mutations were not observed. This finding highlights the fact that there is the absence of universality in the use of pfk13 C580Y, R539T, and Y493H for ART resistance surveillance in all WHO malaria-endemic regions [34]. This assertion is further strengthened after pfk13 M476I was selected for in a Tanzanian clinical isolate in the presence of in vitro ART drug pressure. This suggests the possibility of pfk13 M476I being used as an ART resistance marker in Tanzania and not pfk13 C580Y, R539T, and Y493H [34].

In SEA, an increase in pfpm2 and pfpm3 gene copy number is used as a molecular marker of resistance for PQ in clinical isolates [28]. However, this is not the case in Africa, as high proportions of clinical isolates have multiple copies of the pfmp2 gene which has an association with PQ resistance. For example, more than 30% of clinical isolates from Burkina Faso and Uganda had multiple copies of the pfmp2 gene [35]. The observed high prevalence of multiple gene copies of the pfmp2 gene in African isolates could be that isolates had multiple copies of the gene before introduction of PQ for the treatment of malaria. Therefore, the use of increased gene copy number in pfmp2 and pfpm3 genes as molecular markers of resistance in monitoring DHAP may not be accurate in Africa [35]. The above assertions point to the importance of the identification and validation of peculiar molecular markers of resistance to first-line antimalarial drugs used in a particular country for malaria treatment. This can ensure the accurate use of *Plasmodium* spp. molecular markers of resistance for antimalarial drug efficacy studies in malaria-endemic regions.

## 6. Drug Metabolism in the Human Host

The drug metabolism involves the enzymatic conversion of a therapeutic important chemical into a new molecule inside the human body for a specific activity [21]. The process of the enzymatic conversion may result in pharmacologically active, inactive, or toxic metabolites, depending on the genetic makeup of the individual [21]. The drug metabolic process involves two phases: the conversion of the therapeutic compound into a pharmacological active metabolite by the cytochrome P450 isoenzymes (CYP) and the transport of the pharmacologically active metabolite to their target site of action [21].

## 7. Cytochrome P450 Isoenzymes (CYP) in the Human Host

The main enzymes involved in the antimalarial drug metabolism are the cytochrome P450 (CYP) enzymes. Approximately, 40% of these enzymes are polymorphic. The CYP genes with polymorphisms include CYP1A2, CYP2A6, CYP2B6, CYP2C8, CYP2C9, CYP2C19, CYP3A4, and CYP3A5 [36]. The polymorphisms lead to three main phenotypes, which are classified as poor metabolizers, intermediate metabolizers, and extensive metabolizers. Poor metabolizers break down drugs slowly, which may lead to a more pronounced side effect. Additionally, poor metabolizers might experience treatment failure when administered with prodrugs that need to be bioactivated. Poor metabolizers will have problems in the bioactivation of proguanil to cycloguanil by the CYP2C19 gene [36]. Extensive metabolizers tend to metabolize the drugs more extensively which results in faster relief from the disease symptoms [36]. Intermediate metabolizers metabolize the drugs efficiently, resulting in the optimal concentration of the pharmacologically active metabolite in the plasma, with no toxicity or adverse drug effect being recorded [36].

Polymorphisms in CYP3A4 (the most abundant human CYP enzyme) have a major role in the expression and function of the gene, and this may lead to drug toxicity [37]. In CYP3A5, genetic variation accounts for the majority of its expression and function [36]. In CYP2C8, studies that incubated AQ with human liver microsomes and recombinant expressed CYP2C8 protein from cells observed a 50% reduced metabolic activity for CYP2C8^*∗*^2 and an 85% reduced metabolic activity for CYP2C8^*∗*^3 when compared to the wildtype [38]. For CYP2C19, CYP2C19^*∗*^2 and CYP2C19^*∗*^3 polymorphisms are null alleles which result in the complete absence of protein functions [39]. The CYP2C19^*∗*^17 has been associated with the increased metabolism [40]. Among several polymorphisms in CYP2A6, only CYP2A6^*∗*^2 and CYP2A6^*∗*^7A have reduced 7-hydroxylation of coumarin [41].

## 8. Drug Transport in the Human Host

Transporters are membrane-bound proteins that help in the movement of compounds in and out of cells. Transporters play a very important role in the delivery of metabolized drugs to their target sites [42, 43]. Genetic variations in drug transporter genes in humans are very important in determining the concentration of metabolized drugs in the targeted cells which contribute to the variability of drug response among individuals [42, 44, 45]. The ABCB1 gene which encodes the human MDR1 (P-glycoprotein) protein functions as an efflux transporter and its polymorphic forms ABCB1 c.1236C>T, ABCB1 c.2677G>T/A, and ABCB1 c.3435C>T have been associated with variations in drug availability after the metabolism [42]. The solute carrier organic anion transporter family member 1B1 (SLCO1B1) gene encodes the organic anion transporting polypeptide 1B1 (OATP1B1). The SLCO1B c.521C>T has been associated with an increase in organic anions concentration in plasma by reducing hepatic uptake of organic anions [42, 44]. Also, genetic variations in SLC22A1 and SLC22A2 genes which encode the organic cation transporter proteins OCT1 and OCT2, respectively, influence metformin pharmacokinetics in humans [46–48] (Table 1).

## 9. Typing of Polymorphisms in CYP gene as a Means to Personalize Medication in Malarial Infection: The Setbacks

One of the most effective ways of knowing how an individual will metabolize an antimalarial drug is by genotyping the CYP gene which encodes the enzyme mainly involved in the antimalarial drug metabolism. This makes it an easy approach to personalize medicine. Unfortunately, this is not true for some antimalarial drugs as more than one CYP enzyme can metabolize a single antimalarial drug. For example, piperaquine is metabolized primarily by CYP3A4 and to a lesser extent by CYP2C8 when compared to CYP3A4 [73]. Lumefantrine is metabolized by both CYP3A4 and CYP3A5 [64]. This suggests that mutation(s) in one of the CYP genes leading to a defective metabolism may be compensated for by the second CYP enzyme that can also metabolize the antimalarial drug. Hence, the chances of the poor antimalarial drug metabolism occurring in an individual is less. For some antimalarials such as AQ, both the parent drug and its N-desethlamodiaquine (DEAQ) metabolite are therapeutically active against the malaria parasite. This suggests that AQ can work effectively in the absence of the efficient metabolism by the patients [87, 88]. Due to the functional redundancy in some CYP enzymes and the therapeutical activeness of some parent drugs and their metabolites, it will be important for researchers to focus on the transporters that may play a role in transporting metabolized drugs to their target site of action. How these enzymes contribute to malaria treatment outcomes with the view of improving upon personalized medicine is discussed.

## 10. Sickle Cell Anemia and Malaria

Sickle cell anemia (SCA) is a major health problem in mostly sub-Saharan Africa (sSA) with over 250000 babies born annually with the disease [89]. In Africa, approximately 200000 babies are born with SCA annually and approximately 50% die before the age of five [90]. Individuals with SCA are four times more susceptible to malaria compared with individuals with sickle cell trait. This makes malaria a major contributor to morbidity and mortality in these individuals [90]. Malaria infection in SCA individuals results in severe anemia and painful crises, which can result in the death of these persons. In most malaria-endemic areas, crises due to malaria infection in individuals with SCA occur mostly in high malaria transmission seasons [91]. Due to this knowledge, presumptive malaria treatment is the ideal way of preventing malaria in individuals with SCA. The antimalarial drugs used mostly for presumptive malaria treatment are CQ and SP [92]. Due to the high level of CQ-resistant parasites recorded in most countries in sSA, the use of SP has a higher success rate in preventing malaria in SCA individuals [92]. The antimalarial drug SP is also preferred for presumptive malarial treatment in pregnant women with SCA [93]. The treatment of SCA is mostly by the use of hydroxyurea [94]. The recent use of hydroxyurea for SCA treatment means there is limited data on hydroxyurea and antimalarial drug-drug interactions; hence, the need for investigation in this aspect. Since CQ and SP are mostly used as presumptive treatments for malaria in SCA individuals, it will be ideal for future research to focus on hydroxyurea and CQ or SP drug-drug interactions [92, 94].

## 11. The Use of Genetic Factors of Parasite and Host to Curb Antimalarial Drug Resistance

Detection of *Plasmodium* spp. molecular markers of resistance to antimalarial drugs has proven to be an effective way of identifying potential ADR parasite phenotypes. The use of high throughput sequencing techniques has helped in the identification of molecular markers associated with resistance to antimalarial drugs in efficacy studies in most malaria-endemic countries [2].

The categorization of people by their genotype has proven to be effective in establishing the link between individual pharmacogenetics and antimalarial drug pharmacokinetics [95–97]. This has led to improved drug response in most individuals to antimalarial drugs. This suggests that there is the need to establish a comprehensive worldwide CYP gene polymorphism database, which will incorporate the antimalarial drug pharmacokinetic parameters associated with its CYP gene polymorphism(s) [98]. This will help improve personalized medicine and significantly reduce incidents of adverse drug effects that may be associated with taking antimalarial drugs [21]. For example, pharmacogenetic tests have been used to optimize warfarin doses, avoid tamoxifen treatment failure, and hypersensitivity drug effects associated with abacavir treatment [20]. A similar test can be performed on individuals before the prescription of antimalarial drug for malarial treatment. This will help to ascertain the best antimalarial drug to administer during malarial treatment.

Pharmacogenetic research has become very important due to the possibility of drug-drug interaction, as several drugs such as antiviral, antibacterial, and antimalarial drugs are given in combination to individuals in most malaria-endemic areas. These drugs are substrates, inducers, or inhibitors of CYP enzymes and MDR1 transporters. This makes the chances of drug-drug and/or drug-gene interactions resulting in adverse drug effects highly likely. Due to the abovementioned reasons, there is a need to develop comprehensive clinical data from a large number of patients to assess antimalarial drug pharmacokinetics in relation to dosage and clinical outcomes. The evaluation of individual pharmacogenetics in combination with the *Plasmodium* spp. genetic factors is crucial to ascertain the mechanism of ADR [21]. This assertion is supported by a study conducted by Hodoameda et al. (2020) where it was reported that *P. falciparum* genetic factors rather than host factors are likely to drive resistance to ACT in Ghana, while a study by Kiaco et al. (2017) report that the drug transporter ABCB1 c.3435 C > T SNP influences AL treatment outcome in Angola. Results from both findings highlight the need to factor both the parasite's genetic and host pharmacogenetics in the determination of malaria treatment outcomes. Knowledge of the prevalence of the *Plasmodium* spp. molecular markers of resistance to a particular antimalarial drug can inform policymakers as to which the antimalarial drug should be introduced for use in a particular country. This is also true for the knowledge of the prevalence of pharmacogenetics of individuals in a particular population, as this can help to inform which antimalarial drug will be metabolized effectively by individuals in a population.

## 12. What Is the Major Driver of Antimalarial Drug Resistance between the Factors, Parasite Genetic Factors and Host Pharmacogenetics: The Authors Take

One major puzzle the scientific community wants to bring a final closure to is to ascertain the major driver of antimalarial drug resistance, especially when both the parasite genetic factors and host pharmacogenetics [21] play vital roles in malaria treatment outcomes. Of the two factors, the parasite genetic factor is the major contributor to antimalarial drug resistance [3, 99]. During drug development, one major factor that is considered is the ability of an individual to metabolize the drug efficiently. This ensures that only antimalarial drugs that can be metabolized by the majority of individuals living in malarial endemic regions are developed [21]. Although polymorphism may exist in the CYP genes that can lead to the altered metabolism of a particular antimalarial drug, they are only present at a very low prevalence level in any given population [20, 21, 65]. Additionally, the ability of two or more CYP enzymes to metabolize a particular antimalarial drug results in most antimalarial drugs being metabolized effectively in most individuals [64, 73]. Also, for some antimalarial drugs, both the parent drug and the active metabolite are therapeutically active against the malarial parasite. Due to this, the possibility of the host pharmacogenetics contributing to drug resistance is highly unlikely [3, 64, 73]. For this reason, the major factor which contributes to antimalarial drug resistance is the parasite genetic factor [3, 99]. Mutations in the parasite genome that confers resistance to antimalarial drugs do occur in nature. Although the natural proportion of such mutants is low, they become selected under drug pressure and subsequently become the dominant population over time [3, 100]. Additionally, changes in the parasite genome leading to the selection of resistant parasite strains can occur rapidly due to long exposure to antimalarial drugs. Subsequent redrawer of the antimalarial drug over some time can restore parasite susceptibility to the antimalarial drug [101]. These genetic changes occur in the form of point mutation(s) or increased gene copy number in the antimalarial drug target sites in response to antimalarial drug pressure. Additionally, the rapid spread of resistant parasite strains from one geographical location to the other contributes to global antimalarial drug failure in malaria-endemic regions [3]. The rapid genetic changes in the parasite genome due to drug pressure coupled with the global spread of ADR parasite strains result in antimalarial drug failure in malaria transmission regions within few decades. Hence, the parasite should be the primary focus in our quest to fight antimalarial drug resistance [2]. For this reason, there is the need to constantly search for mutations in the parasite genome to identify possible mutations in antimalarial drug target sites and validate these mutations as molecular markers of resistance or not. This will allow the early detection of resistant parasite strains leading to the rapid implementation of containment strategies to avoid the global spread of resistant parasite strains.

## Figures and Tables

**Table 1:** Summary of current antimalarial drugs, their parasite molecular markers of resistance and human host pharmacogenetics.

Antimalarial drug	Molecular markers of resistance	Cytochrome P450 involved in the metabolism	Transporters involved in the transport of the antimalarial drugs
Quinine	pfmdr1 N86Y, Y184F, S1034C, N1042D, D1246Y [49] pfmrp Y191, A437S [50]	CYP3A4, CYP3A5 [51, 52] CYP2C9 [53]	MDR1 [19] OCT1, OCT2 [47]

Halofantrine	Increased pfmdr1 copy number [54]	CYP3A4 and CYP3A5 [55]	Not available

Mefloquine	pfcrt K76T, A220S, Q271E, N326S, I356T, R371I Increased pfmdr1 copy number, pfmdr1 N86Y [56, 57]	CYP3A4 [58]	MDR1, ABCG2 [59, 60] ABCB1 [61]

Lumefantrine	pfmdr1 N86Y, Y184F, S1034C, N1042D, D1246Y [62] Increased pfmdr1 copy number [63]	CYP3A4 and CYP3A5 [64]	ABCB1 [65].

Chloroquine	pfcrt K76T, K76N, K76I [66] pfmdr1 N86Y [23]	CYP2C8, CYP3A4, and CYP3A5 [67]	The MDR1, MRP1, and MRP4 are involved in the transport of chloroquine [68]

Amodiaquine	pfmdr1 N86Y, Y184F, S1034C, N1042D, D1246Y, pfcrt K72T [69, 70]	CYP2C8, CYP1A1 and CYP1B1 [71]	Not available

Piperaquine	Increased pfpm2 and pfpm3 copy numbers [28, 72]	CYP3A4 and CYP2C8 [73]	Not available

Pyronaridine	pfmdr1 N86Y, Y184F, S1034C, N1042D, D1246Y, pfcrt K72T [74, 75]	CYP1A2, CYP2D6, and CYP3A4 [76]	Not available

Primaquine	Not available	CYP1A2, CYP3A4, and monoamine oxidase [77]	MRD1 and MRP1 [78]

Proguanil	pfdhfr S108N, N51I, and C59R [79]	CYP2C19 and CYP3A4 [21]	MATE1 and MAT2-K [80]

Pyrimethamine	pfdhfr S108N, N51I, C59R, 164 I164L, and A16V [4, 5]	Not available	Not available

Sulfadoxine	pfdhps S436F/A, A437G, K540E, A581G, and A613S/T [4, 5]	Not available	Not available

Artemisinin	pfk13 C580Y, R539T, I543T, F446L, N458Y, P547L, R56IH, Y493H [81], pfatp6 A623E, S769N [82]	CYP2B6, CYP3A4, and CYP2A6 [83]	P-glycoproteins [84]

Atovaquone	pfcytb Y268S/C/N, M133I, L144S, G280D [85, 86]	Not available	Not available
